# The Effects of Using K-Wire or Titanium Elastic Nails on Clinical and Radiological Outcomes in the Métaizeau Closed Reduction Technique

**DOI:** 10.1155/2022/9317837

**Published:** 2022-03-27

**Authors:** Mahmud Aydin, Serkan Surucu

**Affiliations:** ^1^Uğur Mumcu Mahallesi, Belediye Sokak, No. 7 Sultangazi/İSTANBUL, Haseki Education Research Hospital, Istanbul, Turkey; ^2^University of Missouri-Kansas City, Department of Orthopaedic Surgery, Kansas City, USA

## Abstract

**Aim:**

The purpose of this study was to compare the effects of using Kirschner-wire (K-wire) or titanium elastic nails (TEN) on the functional results of patients treated with the Métaizeau closed reduction technique, which is a popular treatment modality for pediatric radial neck fractures (PRNF).

**Methods:**

Patients who underwent the Métaizeau closed reduction technique for PRNF between May 2017 and May 2019 were assessed retrospectively. The Judet classification was used for the PRNF classification. The study consisted of patients who underwent the Métaizeau technique for Judet Type IV PRNFs and had at least 12 months of follow-up.

**Results:**

This study enrolled a total of 43 patients, 22 females and 21 males. Their mean age was 8.69 ± 2.3 at the time of surgery. The mean MEPS of patients undergoing K-wire surgery was 89.50, while the mean MEPS of patients undergoing TEN surgery was 92.83. There was no statistically significant difference between the two groups in terms of MEPS. When the complication rates of the two groups were evaluated, there was no statistically significant difference between them. The cost per patient for TEN was $40. The cost per patient for the K-wire was $1.

**Conclusion:**

The effect of K-wire or TEN used in the Métaizeau closed reduction technique on clinical outcomes is similar, and K-wire is more cost-effective.

## 1. Introduction

Pediatric radial neck fractures (PRNFs) are the most prevalent type of elbow fracture in children aged 9–10 years old and the third most common elbow fracture among all elbow fractures [1, 2]. PRNFs are challenging to treat; a misdiagnosis or a delay in therapy may result in the patient's elbow movements being restricted [3]. Treatment is determined by the angulation of the fracture. Minor degrees of angulation are treated with immobilization only, while displaced fractures are treated with closed reduction or, if reduction cannot be achieved, surgically [4].

The Métaizeau closed reduction technique is now widely used in the surgical management of PRNFs. Titanium elastic nails (TENs) are utilized in the Métaizeau technique for the joystick maneuver during fracture reduction and for fracture stability following reduction [5]. Many studies in the literature have focused on fracture reduction techniques in the Métaizeau closed reduction technique, as well as the comparison of different surgical treatments for PRNFs [6, 7]. However, we were unable to find a study in the literature that investigated the implant features used in the Métaizeau closed reduction technique.

The purpose of this study was to assess functional and radiological outcomes in PRNF patients treated with the Métaizeau closed reduction technique based on the implant type utilized. The study hypothesized that using TEN in the Métaizeau closed reduction technique was not superior to using Kirschner wire (K-wire), and that using K-wire was more cost-effective.

## 2. Methods

### 2.1. Study Design

This study was conducted retrospectively in compliance with the Clinical Research Ethics Committee's ethical criteria and the 1975 Helsinki Declaration, which was revised in 2013. Ethics committee approval was received (Decision No. 2020-217, 25/11/2020). Between May 2017 and May 2019, patients treated for PRNFs were evaluated using hospital digital records and patient files. The Judet classification [8] was used for the classification of PRNF (Table 1). The study enrolled patients who had undergone the Métaizeau technique for Judet Type IV PRNF and were followed for at least 12 months. Patients with nonoperatively treated fractures, less than a 12-month follow-up period, and missing data were excluded. All surgeries were performed by two experienced orthopedic pediatric surgeons.

### 2.2. Surgical Technique

All procedures were carried out under general anesthesia with the use of a tourniquet. To protect the superficial radial nerve, a 1 cm mini-incision was made 2 cm proximal to the growth line at the distal radius laterally. TEN or K-wire were used during the operation, depending on the attending surgeon's preference. The thickness of the implant to be used was determined as two-thirds of the distance measured on the preoperative forearm X-ray by measuring the narrowest region of the medulla of the radius. The K-wire was moved proximally with small rotational motions until it reached the fracture. The surgeon achieves an anatomical reduction of the fracture fragment by lifting it with a percutaneous K-wire, reducing and stabilizing it with rotational movements of the tip of the K-wire and TEN. Following the reduction controlled by intraoperative fluoroscopy, the K-wire, or TEN, was shortened.

### 2.3. Evaluation

For pain treatment and stability, all patients were immobilized in a long arm cast in a neutral position for two weeks. Physical treatment began two weeks later with range-of-motion exercises (passive and active flexibility exercises), followed by forearm strengthening and recovery activities. Eight weeks later, the TEN, or K-wire, was removed in the operating room under general anesthesia. All patients were followed up regularly for 12 months. For radiographic assessment, standard anteroposterior and lateral radiographs were used. To assess functional progress, the Mayo Elbow Performance Score [9] (MEPS) was used.

### 2.4. Statistical Analysis

Statistical analyses were performed using SPSS software (version 20.0; IBM Corp., Armonk, NY, USA). Descriptive statistics were expressed as means, medians, standard deviations, ranges, and percentages. The data were tested for normality using the Kolmogorov–Smirnov test. A comparative analysis of two independent groups was performed using Pearson's chi-square test for categorical variables. An independent samples *t*-test was performed for continuous variables by normality testing. A two-sided *p* < 0.05 was considered significant. By assuming the difference in the large effect size between the groups (effect size = 0.55), the sample size was calculated as 95% power and 43 cases for the alpha significance level of 0.05.

## 3. Results

A total of 43 patients, 22 girls and 21 boys, were included in this study. The mean age at surgery was 8.69 ± 2.3 years (range, 5–12 years). Both groups were comparable in terms of demographic and clinical characteristics (Table 2). All patients were followed for a minimum of 12 months (mean 17.1 ± 3.76 months; range, 12–24 months). Fracture union was achieved in all patients.

Two of the 20 patients who underwent K-wire surgery were found to have posterior interosseous nerve (PIN) deficiency, and one had radial head avascular necrosis. One of the 23 patients who had TEN had a PIN deficiency, and one had radial head avascular necrosis. The PIN deficiency was managed nonoperatively and resolved spontaneously within six months. There was no statistically significant difference between the two groups in terms of complication rates (*p*=0.760).

The mean MEPS of patients who underwent K-wire surgery was 89.50, while the mean MEPS of patients who underwent TEN surgery was 92.83. There was no statistically significant difference in MEPS between the two groups (*p*=0.221).

When the fracture types of the patients who were operated on with K-wire were assessed, 16 (80%) of the 20 patients had Judet Type 4a fractures and 4 (20%) had Judet Type 4b fractures. When the fracture types of the patients who were operated on with TEN were assessed, 9 (39.1%) of the 20 patients had Judet Type 4a fractures and 4 (60.1%) had Judet Type 4b fractures. In terms of fracture types, there was a statistically significant difference between the two groups (*p*=0.008).

When we evaluated the cost-effectiveness, the TEN® (TST medical devices, Turkey) cost $40 per patient, while the K-wire (Academy medical instruments, Turkey) cost $1 per patient. Métaizeau technique was 98% cheaper when K-wire was used.

## 4. Discussion

The degree of angulation of the fracture determines the treatment plan for PRNFs. Judet Type 1 and 2 fractures can be treated nonoperatively with short-term immobilization. The majority of these individuals have a good prognosis and functional outcomes [6]. The treatment of Judet Type 3 radial neck fractures remains controversial in the literature [10]. For Judet Type 4 radial neck fractures, surgical treatment is recommended [11]. By including only Judet Type 4 fractures in our study, we eliminated any effect of fracture type on treatment outcomes. Additionally, we used the Métaizeau closed reduction technique, which has gained popularity in recent years [12, 13], as a surgical technique.

There is a study comparing TEN and K-wire in the surgical treatment of various fractures [14]. Although studies describe and compare surgical methods for the treatment of PRNFs in the literature [12, 13, 15], there is no study evaluating the properties of the implant in the Métaizeau closed reduction technique. In this study, we evaluated the outcomes of patients who underwent surgery with the Métaizeau closed reduction technique due to Judet Type 4 PRNF based on the characteristics of the implant utilized during the surgery.

In this study, there was no statistically significant difference in MEPS between the two groups. The functional outcomes of the TEN and K-wire groups were comparable. There was no statistically significant difference in complication rates between the TEN and K wire groups. At the same time, the radiographic and functional outcomes of the patients in this study were consistent with previously published studies [13, 16, 17]. When we analyzed the costs of the two groups, we found significant differences. TEN was 40 times more expensive than K wire. In addition, the fact that the K wire is the basic material in the orthopedic operating room makes it more advantageous than TEN.

This study has some limitations. The significant difference between the two groups in terms of Judet Type 4 fracture subgroups may affect the results. In addition, another limitation is that the study is retrospective. Biomechanical studies can be carried out to reveal the biomechanical differences of the use of K wire and TEN in PRNFs.

## 5. Conclusion

After analyzing the outcomes of this study and the cost-effectiveness of both treatment choices, we conclude that K-wire use is a cost-effective treatment option that produces comparable results. Osteosynthesis with closed intramedullary K-wire may be a realistic choice for the treatment of pediatric radius neck fractures in developing countries.

## Figures and Tables

**Table 1:** Judet classification.

Type	Angulated angle (°)	Displacement (%)
I	No	No
II	>30	<1/2 of transverse diameter
III	30–60	>1/2 of transverse diameter
IV	>60	Total
Judet IVa: angulated angle of 60–80°; Judet IVb: angulated angle >80°		

**Table 2:** Comparison of the outcomes of two methods for the treatment of Judet type IV radial neck fractures.

Surgical technique		Métaizeau closed reduction technique with K-wire	Métaizeau closed reduction technique with TEN	*p* value
Age mean ± SD median (min–max)		8.78 ± 2.20 (5–12)	8.65 ± 1.85 (5–11)	0.875^∗^

Judet classification	Judet type 4A	16 (80%)	9 (39.1%)	0.008^∗^
	Judet type 4B	4 (20%)	14 (60.9%)	

MEPS mean ± SD		89.5 ± 8.57	92.83 ± 8.90	0.221^∗∗^
Median (min_max)		(85) (70–100)	(100) (70–100)	

Complication	PIN deficit	2 (10%)	1 (4.3%)	0.760^∗^
	Avascular necrosis	1 (5%)	1 (4.3%)	
	None	17 (85%)	21 (91.3%)	

PIN; posterior interosseous nerve, TEN; titanium elastic nail. MEPS; mayo elbow performance score, K-wire; Kirschner-wire. ^∗^Independent samples *t*-test. ^∗∗^Pearson's chi-square test.

## Data Availability

The data were collected using hospital digital records and patient files.

## Additional Points

*What's Known*. (i) Pediatric radial neck fractures are the third most common elbow fracture among all elbow fractures. (ii) The Métaizeau closed reduction technique is the most popular method for the surgical treatment of pediatric radial neck fractures. (iii) Titanium elastic nails (TENs) are used for stability and reduction in the Métaizeau technique. *What's New*. (i) In this study, we evaluated the results of patients who were operated with the Métaizeau closed reduction technique according to the characteristics of the implant used. (ii) TEN was 40 times more expensive than K-wire.
